# Strained Si_0.2_Ge_0.8_/Ge multilayer Stacks Epitaxially Grown on a Low-/High-Temperature Ge Buffer Layer and Selective Wet-Etching of Germanium

**DOI:** 10.3390/nano10091715

**Published:** 2020-08-29

**Authors:** Lu Xie, Huilong Zhu, Yongkui Zhang, Xuezheng Ai, Guilei Wang, Junjie Li, Anyan Du, Zhenzhen Kong, Xiaogen Yin, Chen Li, Liheng Zhao, Yangyang Li, Kunpeng Jia, Ben Li, Henry H. Radamson

**Affiliations:** 1 Key Laboratory of Microelectronics Devices & Integrated Technology, Institute of Microelectronics, Chinese Academy of Sciences, Beijing 100029, China; xielu@ime.ac.cn (L.X.); zhangyongkui@ime.ac.cn (Y.Z.); aixuezheng@ime.ac.cn (X.A.); lijunjie@ime.ac.cn (J.L.); duanyan@ime.ac.cn (A.D.); kongzhenzhen@ime.ac.cn (Z.K.); yinxiaogen@ime.ac.cn (X.Y.); lichen2017@ime.ac.cn (C.L.); zhaoliheng@ime.ac.cn (L.Z.); liyangyang@ime.ac.cn (Y.L.); jiakunpeng@ime.ac.cn (K.J.); 2 Microelectronics Institute, University of Chinese Academy of Sciences, Beijing 100049, China; 3 Research and Development Center of Optoelectronic Hybrid IC, Guangdong Greater Bay Area Institute of Integrated Circuit and System, Guangzhou 510535, Guangdong, China; liben@ime.ac.cn; 4 School of Microelectronics, University of Science and Technology of China, Hefei 230026, Anhui, China

**Keywords:** vertical Gate-All-Around (vGAA), high-quality Si_0.2_Ge_0.8_/Ge stack, selective etching of germanium

## Abstract

With the development of new designs and materials for nano-scale transistors, vertical Gate-All-Around Field Effect Transistors (vGAAFETs) with germanium as channel materials have emerged as excellent choices. The driving forces for this choice are the full control of the short channel effect and the high carrier mobility in the channel region. In this work, a novel process to form the structure for a VGAA transistor with a Ge channel is presented. The structure consists of multilayers of Si_0.2_Ge_0.8_/Ge grown on a Ge buffer layer grown by the reduced pressure chemical vapor deposition technique. The Ge buffer layer growth consists of low-temperature growth at 400 °C and high-temperature growth at 650 °C. The impact of the epitaxial quality of the Ge buffer on the defect density in the Si_0.2_Ge_0.8_/Ge stack has been studied. In this part, different thicknesses (0.6, 1.2 and 2.0 µm) of the Ge buffer on the quality of the Si_0.2_Ge_0.8_/Ge stack structure have been investigated. The thicker Ge buffer layer can improve surface roughness. A high-quality and atomically smooth surface with RMS 0.73 nm of the Si_0.2_Ge_0.8_/Ge stack structure can be successfully realized on the 1.2 µm Ge buffer layer. After the epitaxy step, the multilayer is vertically dry-etched to form a fin where the Ge channel is selectively released to SiGe by using wet-etching in HNO_3_ and H_2_O_2_ solution at room temperature. It has been found that the solution concentration has a great effect on the etch rate. The relative etching depth of Ge is linearly dependent on the etching time in H_2_O_2_ solution. The results of this study emphasize the selective etching of germanium and provide the experimental basis for the release of germanium channels in the future.

## 1. Introduction

In recent years, with the continuous scaling-down of CMOS technology nodes, high-mobility channel materials (SiGe, Ge and III–V material such as InGaAs) and novel device designs (horizontally/vertically Gate-All-Around (GAA) stacked nanowires) have been under investigation [1,2,3,4,5,6,7,8,9,10,11,12,13]. The high mobility of n-GaN and the current possibility of achieving an enhancement mode in non-polar GaN have also been extensively researched with gallium nitride Fin Field-Effect Transistors (FinFETs) [14,15]. For the channel material, Ge can be grown on Si and is a promising material with the advantage of having a higher carrier mobility compared to Si, InAs and GaAs [16]. The growth of a high-quality Ge layer on Si is one of the main challenges in achieving a vertical GAA structure. So far, many studies have proposed Si/SiGe multilayer structures to create an SiGe channel layer [17,18,19,20,21]. In these studies, the solutions of TMAH, mixture solution HF:H_2_O_2_:CH_3_COOH and other alkaline solutions have often been used to obtain a SiGe channel [22,23,24,25]. However, in the traditional FinFET structure, an important problem is the dry-etching damage to the sidewall caused by plasma sputtering in the fin formation process [26,27,28,29]. Therefore, the wet-etch method is advantageous for the formation of advanced GAA stacked nanowires.

Use of SiGe/Ge multilayers with Ge as a channel layer and proper selective etching with enough accuracy to reach beyond 3 nm channel length have not been properly studied yet. Since vertical GAA transistors are considered one of the potential devices for the next era of CMOS technology, it is crucial to design a gate and control channel size. Therefore, it is necessary to study new structures (e.g., SiGe/Ge multilayers), and the selective etching of a channel Ge layer is an important issue to be investigated. In this work, we have studied the complete process of the formation of a 20 nm Ge channel layer in Vertical Gate-All-Around (VGAA) NWs, including the epitaxy and etching processes, with a focus on the selective etching of Ge relative to SiGe in Si_0.2_Ge_0.8_/Ge multilayers with HNO_3_ and H_2_O_2_ solution.

## 2. Materials and Methods

In this study, 8 inch p-type Si (100) wafers with resistivity of 8–12 ohm·cm were processed for the test structures. The SiGe/Ge thin films were grown in a reduced pressure chemical vapor deposition (RPCVD) reactor using dichlorosilane (SiH_2_Cl_2_) and germane (10% GeH_4_ in H_2_) as precursors for Si and Ge, respectively. The thicknesses of the SiGe layers were kept below the critical thickness published for SiGe/Ge systems [30,31]. 

A two-step low-/high-temperature (LT-HT) growth was performed to form the Ge buffer layer. At first, a Ge seed layer was deposited at 400 °C, and a high-quality Ge film was subsequently grown at 650 °C. A post-growth in situ annealing was applied at 820 °C in ambient H_2_ to reduce the threading defect density (TDD) below 10^6^ cm^−2^. Finally, the Si_0.2_Ge_0.8_/Ge multilayer stacks were grown at 500 °C with different thicknesses. The low growth temperature was chosen to avoid intermixing of SiGe layers with Ge. 

**Figure 1:**
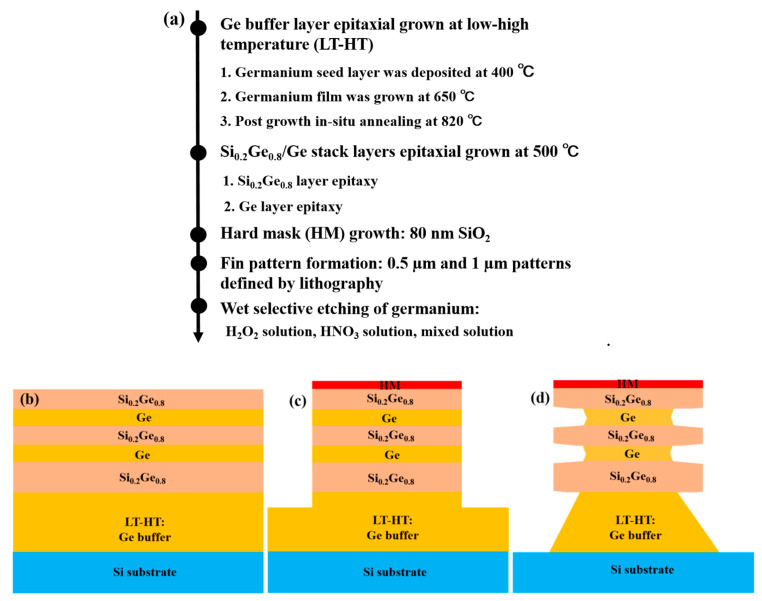
(**a**) Main process flow diagram, (**b**) material structure, (**c**) fin pattern formation with hard mask (HM), (**d**) wet selective etching of Ge in Si_0.2_Ge_0.8_/Ge multilayers.

The fins were formed by depositing an oxide hard mask (HM), and the formed pattern was dry-etched by lithography. At this stage, the samples were cut into small slices to facilitate etching experiments. The wet selective etching of Ge in the Si_0.2_Ge_0.8_/Ge multilayers was carried out at room temperature in mixed solutions of hydrogen peroxide (H_2_O_2_), nitric acid (HNO_3_) and 20%HF/30%H_2_O_2_/99.8%CH_3_COOH at a volume ratio of 1:2:3. During the experiment, slices reduced die error. Slicing makes the most of the wafer and minimizes the inter-chip operating error. Before the etching experiment, all samples were immersed in BOE (49wt% HF and 40wt% NH_4_F with volume ratio of 1:7) for 5 min to remove the natural oxide. As high HF concentrations can damage Ge or SiGe, the BOE solution was mixed with deionized water at a ratio of 1:100, and the samples were cleaned in deionized water for 2 min after etching. Figure 1a–d displays the main process flow and the manufacturing steps of fins and the Ge channel layer in this study. 

Cross-section morphology and etching profile of the experimental samples were analyzed by scanning electron microscopy (SEM). The samples were also characterized by transmission electron microscopy (TEM) to determine crystalline quality, layer profile and to evaluate the results of dry and wet-etching. Furthermore, energy-dispersive spectroscopy (EDS) was employed to determine the element materials of etched layers. Atomic force microscopy (AFM) was used to measure surface roughness. High-resolution X-ray diffraction (HRXRD) and high-resolution reciprocal lattice maps (HRRLMs) were used to measure the strain relaxation, interface and layer quality of SiGe/Ge multilayer structures. 

## 3. Results and Discussion

### 3.1. High Epitaxial Quality of the Si_0.2_Ge_0.8_/Ge Stack Structure

There were three epitaxial samples with Ge buffer layers of varying thickness, among which sample A had a Ge buffer layer thickness of 0.6 µm, sample B 1.2 µm and sample C 2.0 µm. For these samples, the stack layer thicknesses of Si_0.2_Ge_0.8_/Ge/Si_0.2_Ge_0.8_/Ge/Si_0.2_Ge_0.8_ were kept to 122/20/75/20/75 nm. 

**Figure 2:**
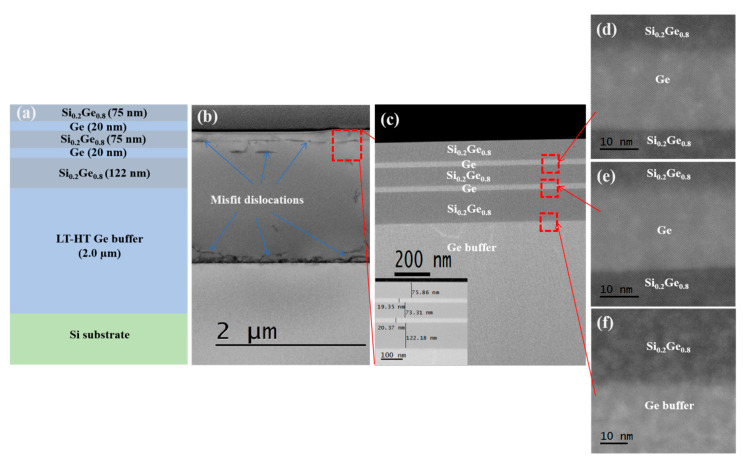
(**a**) The epitaxial stack structure diagram of Si/Ge buffer/Si_0.2_Ge_0.8_/Ge; (**b−c**) TEM images for the Si_0.2_Ge_0.8_/Ge multilayers grown on a 2.0 µm Ge buffer layer (sample C); the inset in (**c**) shows the thickness of each layer determined by TEM; (**d−f**) high-resolution images at interface between Si_0.2_Ge_0.8_ and Ge.

In order to analyze the crystal quality of the epitaxial layer and further measure the layer thickness, TEM is needed. Figure 2 presents TEM analysis of the Si_0.2_Ge_0.8_/Ge stack structure with a 2.0 µm Ge buffer layer on the Si substrate. As can be seen from Figure 2b, the threading dislocation (TD) defects were confined to the Ge buffer layer. In addition, it can be seen from Figure 2c that a high-quality Si_0.2_Ge_0.8_/Ge stack was successfully prepared with minor TD defects. It is also clearly seen that the stack structure contained a smooth surface and abrupt interfaces. The inset in Figure 2c shows the thicknesses of the epitaxial layers obtained by analyzing the TEM images directly, which agreed perfectly with the designed values. We can see that there are sharp Si_0.2_Ge_0.8_/Ge interfaces and rough Ge/Si_0.2_Ge_0.8_ interfaces in Figure 2d−f. This is because during the growing of heterostructures, an ordered edge misfit dislocations (MDs) grid is formed at the interfaces. The edge MDs of a rough Ge/Si_0.2_Ge_0.8_ interface that rapidly formed are sessile dislocations, which cannot penetrate through the buffer layer to the Ge buffer/Si interface by means of gliding [32]. 

**Figure 3:**
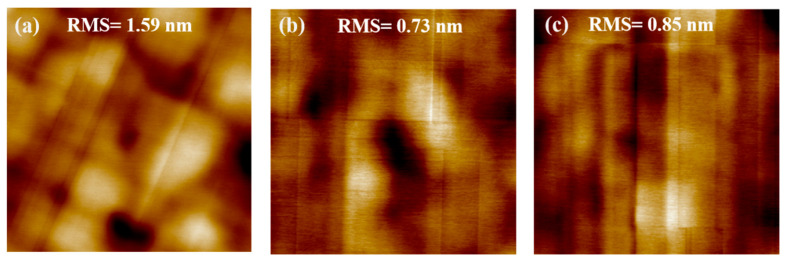
AFM images (10 × 10 μm^2^) of Si_0.2_Ge_0.8_/Ge multilayers grown on (**a**) 0.6, (**b**) 1.2 and (**c**) 2.0 µm thick Ge buffer layers. The estimated RMS for surface roughness values of these samples are 1.59, 0.73 and 0.85 nm, respectively.

Figure 3 shows the AFM images from Si_0.2_Ge_0.8_/Ge stack layers grown on the Ge buffer layer with various thicknesses. The root mean square (RMS), which is the indicator for roughness, decreased from 1.59 nm to 0.73 nm with increasing thickness of Ge buffer layers from 0.6 µm to 1.2 µm. However, as the germanium thickness increased to 2.0 µm, the surface roughness was similar to that of 1.2 µm. The roughening of Si_0.2_Ge_0.8_/Ge stack layers grown on thin Ge buffer layers was mainly due to the formation of misfit dislocations with crosshatch undulation [33], surface diffusion and intermixing of Ge and Si at high temperature (650 °C) during the growth of the Ge buffer layer [34]. As can be seen from the analysis above, the thick Ge buffer layer reduced the surface roughness of Si_0.2_Ge_0.8_/Ge stack layers. Increasing the thickness of the Ge buffer layer can effectively improve the surface roughness.

**Figure 4:**
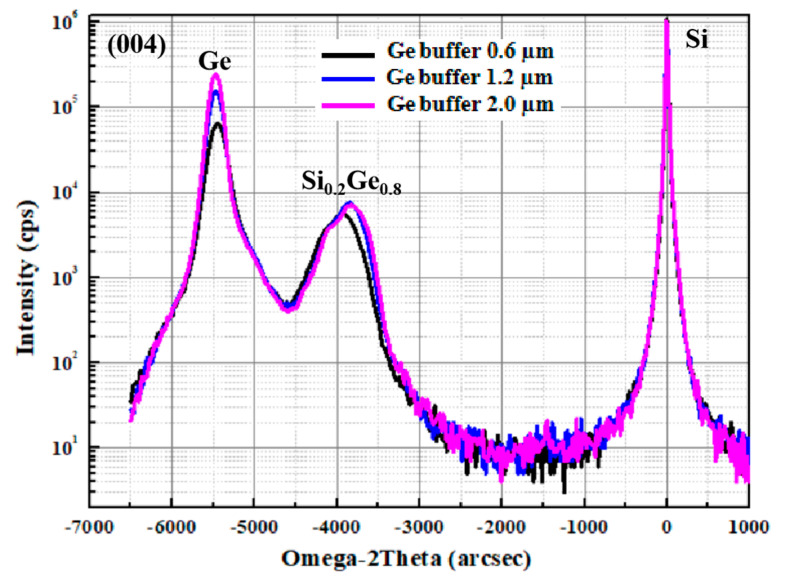
HRXRD spectra measured with various Ge buffer layer thicknesses of 0.6, 1.2 and 2.0 µm. The FWHM values of Ge peaks are 210 arcsec (sample A), 165 arcsec (sample B) and 150 arcsec (sample C), respectively.

In order to further evaluate the structure and quality of epitaxial samples, HRXRD analysis scanning around the (004) diffraction order was implemented on the Si_0.2_Ge_0.8_/Ge stack structure with various Ge buffer thickness. The HRXRD results are shown in Figure 4. For the 0.6 µm Ge buffer layer (sample A), a broadened Ge peak in the omega direction (incident angle) was observed with a high full-width at half maximum (FWHM) value of 210 arcsec. The FWHM values decreased to 165 arcsec with the thicker Ge buffer layer (1.2 µm). When the thickness of the germanium buffer layer was 2.0 µm, the Ge peak had a higher intensity and a lower FWHM value (about 150 arcsec) than those of the thinner buffer layer. It can be seen that the thickening of the buffer layer can enhance the crystal quality of the germanium layer and inhibit the continuous upward extension of defects, laying a foundation for the growth of the subsequent film layer.

In the HRXRD spectrum, FWHM characterizes the crystal quality, and the broadening of the diffraction peak indicates an increase in the surface roughness. These results confirm that the thick Ge buffer layers were almost completely relaxed relative to the Si substrate, and the strain Si_0.2_Ge_0.8_ layers were grown on the Ge layer, as no small-intensity fringes were found. Figure 4 also shows that the peak value of the Si_0.2_Ge_0.8_ layer shifted to right as the buffer thickness increased, which shows that the SiGe layers are more strained. It can be noted that good crystalline quality Si_0.2_Ge_0.8_/Ge stack layers can be achieved with the thick Ge buffer layer because the peaks of Si_0.2_Ge_0.8_ and Ge had almost no fringes on each side. Thicker Ge buffers can increase the quality of Si_0.2_Ge_0.8_/Ge stack layers, but 2 µm Ge results in an increase of substrate bow [35]. Therefore, the flat surface of the 1.2 µm Ge buffer layer can meet the requirement of Ge channel of vertically stacked nanodevices. 

**Figure 5:**
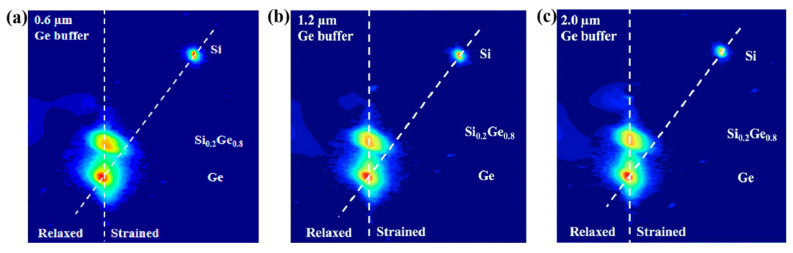
HRRLMs around (113) reflection of strained Si_0.2_Ge_0.8_/Ge stack layers grown on a Ge buffer layer with thicknesses of (**a**) 0.6, (**b**) 1.2 and (**c**) 2.0 µm.

High-resolution reciprocal lattice maps (HRRLMs) around (113) reflection have been performed to analyze the strain of Si_0.2_Ge_0.8_/Ge stack layers grown on Ge buffer layers with different thicknesses, as shown in Figure 5. It is further confirmed that the thick Ge buffer layers were highly relaxed, and the strained Si_0.2_Ge_0.8_/Ge stack layered epitaxially on the Ge buffer/Si (100) layer. From these measurements, we obtained an Si content in the SiGe layer around 20%. The epitaxial layers on the thick Ge buffer layer can be achieved with high film quality and strain amount. Considering their AFM, XRD and HRRLM results, the Si_0.2_Ge_0.8_/Ge stack structures grown on the 1.2 µm Ge buffer layer was the best choice. Therefore, the rest of the experiments were carried out on the stack structure with a 1.2 µm Ge buffer layer. 

### 3.2. Selective Wet-Etching of Ge to Si_0.2_Ge_0.8_

In order to fabricate a germanium channel in Gate-All-Around vertical nanowires (NWs), Ge needs to be laterally released. Therefore, studies in this section are focused on the selective etching of Ge to SiGe. For the samples used in the experiments, the structure profile was kept constant in thickness and composition as follows: 120 nm Si_0.2_Ge_0.8_/35 nm Ge/75 nm Si_0.2_Ge_0.8_/35 nm Ge/75 nm Si_0.2_Ge_0.8_. It is well known that germanium is more active than silicon, and its oxide GeO_2_ is soluble in water, with solubility 0.4g/100mL; hence, germanium is soluble in strong acids and bases. In this paper, the effects of different solutions, including acids (HNO_3_ and H_2_O_2_) and acid mixtures of 20%HF/30%H_2_O_2_/99.8%CH_3_COOH (1:2:3), on lateral etching of germanium were studied.

**Figure 6:**
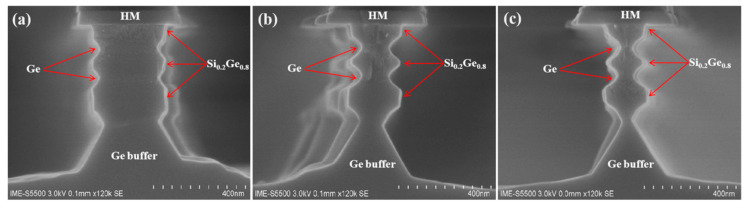
The SEM cross-section images of the Ge buffer/Si_0.2_Ge_0.8_/Ge stack structure after etching at different times in mixed solution HF/H_2_O_2_/CH_3_COOH (1:2:3): (**a**) etching for 2 s, (**b**) etching for 4 s, (**c**) etching for 6 s.

Figure 6 shows the SEM images of the cross-section profile after etching with a mixed solution of HF/H_2_O_2_/CH_3_COOH at different etching times. The results show that the etching rate of Si_0.2_Ge_0.8_/Ge stack layers in mixed solution was fast (about 25 nm/s in Si_0.2_Ge_0.8_ and 37 nm/s in Ge), and Ge had a very low selection ratio to Si_0.2_Ge_0.8_. The layer boundaries of multilayer structures were not obvious, but we could judge the elements in each layer by the amount of etching. The pyramidal shape indicated the Ge (111) plane had a higher etch rate than the Ge (110) plane [36]. Due to time, the etching repeatability of the mixed solution was poor, and the etching rate was difficult to control.

Another solution for etching germanium is nitric acid. HNO_3_ solution, especially at a high concentration, is easy to volatilize. A light agitation causes a change in concentration and temperature, which can greatly affect our experimental results. Thus, all solutions were carefully designed, and the concentration was measured at regular intervals with a nitric acid concentration measuring instrument to ensure that our experiment was carried out in a stable state. When a high concentration of nitric acid is diluted with deionized water, the temperature will increase, so the experiment should be done after the solution is sealed, and it should be placed for 24 h until it reaches room temperature.

**Figure 7:**
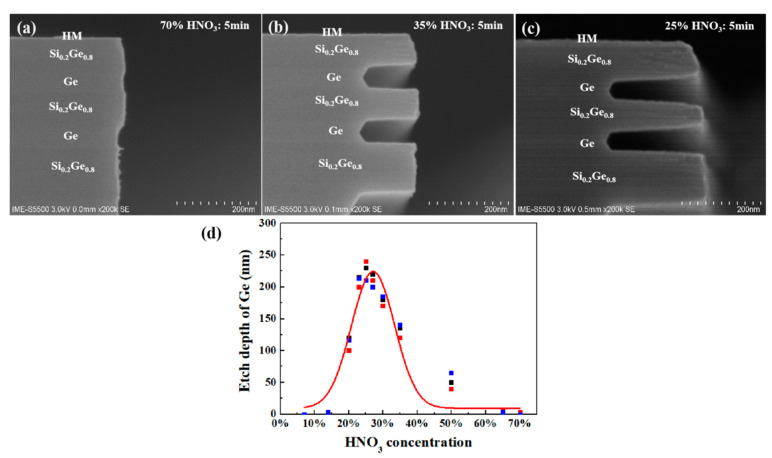
The SEM cross-section images of the Ge buffer/Si_0.2_Ge_0.8_/Ge stack structure in HNO_3_ solution for 5 min in different concentrations: (**a**) 70% HNO_3_, (**b**) 35% HNO_3_ and (**c**) 25% HNO_3._ (**d**) Etching rate of germanium in HNO_3_ solution as a function of HNO_3_ concentration.

The SEM images of selectively etched germanium in the Ge buffer/Si_0.2_Ge_0.8_/Ge stack structure with HNO_3_ solution are in Figure 7. The samples were immersed in nitric acid with different concentrations for 5 min. Figure 7a shows that Ge was almost not etched with nitric acid at the concentration of 70%, mainly playing an oxidant role. With the decreasing concentration of nitric acid solution, the etching rate became faster. At the concentration of 35%, there was a relative etching amount of Ge of 135 nm. When the nitric acid concentration was as low as 25%, 230 nm Ge was relatively etched. It can be seen that the etching of germanium by nitric acid increased gradually with the decrease of concentration. Ge had a high etching selection ratio to Si_0.2_Ge_0.8_. However, it can be seen from Figure 7d that when the concentration of nitric acid was lower than 27%, the etching rate decreased with the decrease of concentration, and it had no effect on germanium when the nitric acid concentration was as low as 7%. The etching rate of germanium in HNO_3_ solution is a function of HNO_3_ concentration and is also affected by stirring rate and temperature [37]. The reaction mechanisms of concentrated and diluted nitric acid to germanium are different, as shown in the following equation. Formula (1) shows the reaction with a high concentration of nitric acid to germanium. HNO_3_ solution mainly acts as an oxidant, and the reaction product is GeO_2_·H_2_O. In the whole reaction process, enough GeO_2_ covers the entire germanium surface to produce passivation and prevent further etching of germanium. The reaction of dilute nitric acid with germanium can be seen in Formula (2). The reaction product is H_2_GeO_3_, which is easy to peel from the germanium surface into the solution.

Concentrated nitric acid:

(1) $$Ge+4HNO_{3}\to GeO_{2}\cdot H_{2}O+4NO_{2}↑+H_{2}O$$

Dilute nitric acid:

(2) $$3Ge+4HNO_{3}+H_{2}O\to 3H_{2}GeO_{3}+4NO↑$$

It is well known that hydrogen peroxide etches germanium. As already mentioned, the high etch rates of Ge are related to the high solubility of GeO_2_ in water. Generally, it is assumed that etching occurs by the oxidation of germanium by H_2_O_2_, followed by dissolution of the oxidation products in aqueous solution, which can be seen in the following Equations (3)–(5):

(3) $$Ge+H_{2}O_{2}\to \mathrm{GeO}+H_{2}O$$

(4) $$GeO+H_{2}O_{2}\to \mathrm{Ge}O_{2}+H_{2}O$$

(5) $$GeO_{2}+H_{2}O\to H_{2}\mathrm{Ge}O_{3}$$

**Figure 8:**
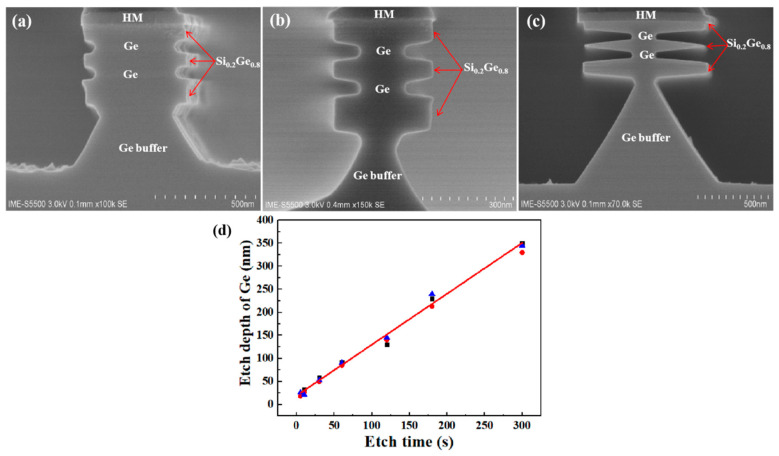
The Ge buffer/Si_0.2_Ge_0.8_/Ge stack structure immersed in H_2_O_2_ (30%) solution for different etching times: (**a**) 30 s, (**b**) 1 min, and (**c**) 5 min. (**d**) The relative etching depth of Ge in H_2_O_2_ solution is linear with the etching time.

The etching profiles of Ge buffer/Si_0.2_Ge_0.8_/Ge stack layers immersed in H_2_O_2_ (30%) solution for different etching times are shown in Figure 8. Among them, Figure 8a shows Ge was etched by 58 nm relative to Si_0.2_Ge_0.8_ with etching time of 30 s. Figure 8b,c shows the relative etching amounts of Ge were 92 nm and 350 nm when the etching times were 1 min and 5 min, respectively. Figure 8d shows that the relative etching depth of Ge was linear with etching time. It can be seen that the H_2_O_2_ solution also etched the Si_0.2_Ge_0.8_ layer during the experiment, which we need to avoid. Germanium was etched at any H_2_O_2_ concentration, even at 0.1%, and with the decrease of H_2_O_2_ concentration, the etch rate slowed down, and the etch morphology became very poor (not shown in this paper).

**Figure 9:**
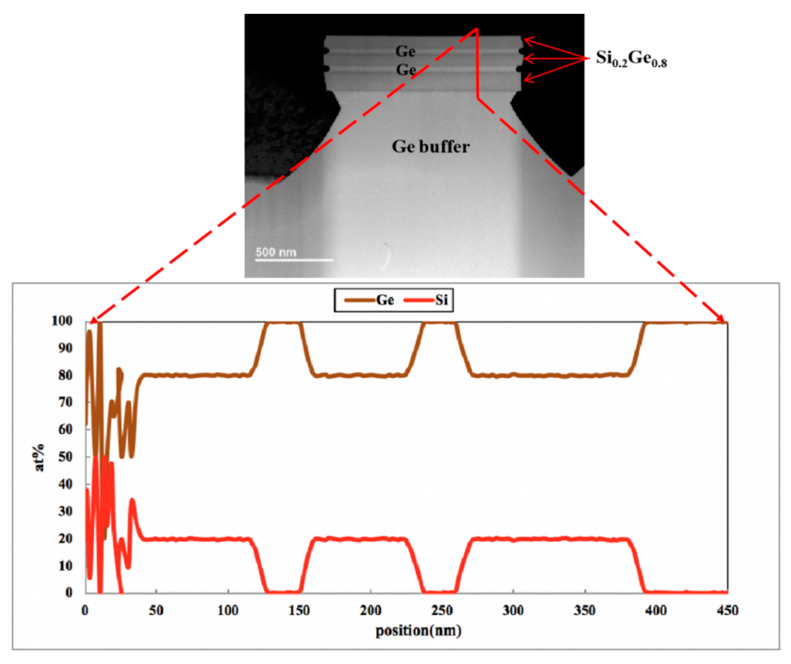
TEM image of etching profile and EDS analysis with line scanning of Si and Ge in vertical orientation of the Ge buffer/Si_0.2_Ge_0.8_/Ge stack structure.

The epitaxial layer quality and more detailed information about the selectivity of different parts of the Si_0.2_Ge_0.8_/Ge stack structure were obtained by HRTEM and EDS. Figure 9 displays the TEM micrograph cross-sections and element analysis at areas of the sample etched with H_2_O_2_ solution (etched for 10 s). The result shows that Ge content in the SiGe layer was about 80%, which is consistent with our analysis above, and there was no mixing between the layers.

**Figure 10:**
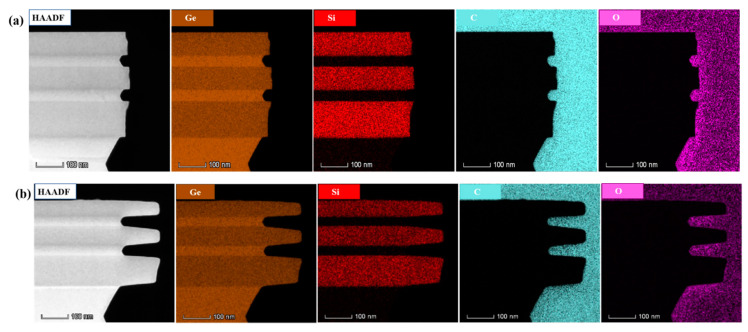
EDS mapping near etching regions with elements Ge, Si, C and O, (**a**) sample etching for 10 s with H_2_O_2_ solution, (**b**) sample etching for 2 min with H_2_O_2_ solution.

Figure 10 shows the element analysis at areas of the sample etched for (a) 10 s and (b) 2 min with H_2_O_2_ solution. The results show that the boundary of each layer was consistent with the design structure, and there was no obvious diffusion of elements. The results provide an experimental basis for the release and selective etching of germanium channels in the future.

## 4. Conclusions

High-quality strained Si_0.2_Ge_0.8_/Ge multilayer structures have been grown on an LT-HT Ge buffer layer and processed with wet-etching of Ge with high selectivity to SiGe. We compared the effects of Ge buffer layers with various thicknesses (0.6, 1.2 and 2.0 µm) on the quality of Si_0.2_Ge_0.8_/Ge stack layers. The thicker Ge buffer layer can improve surface roughness. A high quality and atomically smooth surface with RMS 0.73 nm of the Si_0.2_Ge_0.8_/Ge stack structure can be successfully realized on the 1.2 µm Ge buffer layer. In order to release germanium, the Ge in Si_0.2_Ge_0.8_/Ge multilayers can be easily etched with good selectivity using HNO_3_ and H_2_O_2_ solution at room temperature. It has been found that the solution concentration has a great effect on the etch rate. The etching rate of germanium in HNO_3_ solution is a function of HNO_3_ concentration, and the relative etching depth of Ge is linear with etching time in H_2_O_2_ solution.
